# Editorial: What Is Wrong With Leader Emergence?

**DOI:** 10.3389/fpsyg.2022.884629

**Published:** 2022-05-20

**Authors:** Zeynep Aycan, Mustafa F. Ozbilgin, Kim Yin Chan

**Affiliations:** ^1^Department of Psychology, Koç University, Istanbul, Turkey; ^2^Faculty of Management, Koç University, Istanbul, Turkey; ^3^Brunel Business School, Brunel University London, London, United Kingdom; ^4^Nanyang Business School, Nanyang Technological University, Singapore, Singapore

**Keywords:** emergence, effectiveness, agency, competence, leadership

“Who tends to become leaders or assume leadership roles?” and “Are leaders born or made?”—these questions drove research interest on leader emergence (LE) that began at a time of Great Man theory when the assumption was that certain individuals were pre-destined to lead. These questions led to trait, behavioral, contingency, social-cognitive, relational and management research on LE over the last century. Interest in LE continues today, driven by organizational needs for managerial selection and real-world concerns for social change. Concerned with the rise of authoritarian leaders during WWII, Northway (1946) asked: “how do leaders emerge and more especially, how do the demagogues and charlatans, of whom we are now world weary, arise?” (p. 190). Half a century later, Hogan et al. (1994) used LE research findings to explain “Why are there so many flawed leaders?” Today, researchers continue to explain why dominant, authoritarian leaders seem to attract follower support in times of uncertainty despite the presence of other respectable, “prestige” candidates (e.g., Kakkar and Sivanathan, 2017; see also, Harms et al., 2018). Samdanis and Özbilgin (2020) remind us that even *atypical* leaders are naively trusted to nominate successors who would promote workforce diversity and workplace democracy.

“What's wrong with LE?” was our tongue-in-cheek recognition that the past century's social scientific journey focused on LE in social settings did in fact produce important insights that challenged the originating questions and their assumptions. Besides the 1980s realization that leader emergence and leader effectiveness are two different criteria of leadership with different levels of measurement (cf. Lord et al., 1986), social psychological LE research (e.g., Hollander, 1961) also contributed to today's basic understanding that “leadership is not only about the leader”, that leadership concerns collective, shared and relational processes that are not solely dependent on individual leader emergence or actions. Hanna et al. (2021) recently called for more multilevel study of leadership emergence in organizations to incorporate individual LE processes with the unit-level dynamics and emergent states.

Researchers like Acton et al. (2019) have raised concerns with the lack of conceptual and operational clarity and the apparent incoherence of LE research, largely dominated by the quantitative correlational and experimental research traditions of scientific psychology. While some studies operationalize LE via objective indicators like leadership role occupancy, others use proxies like nomination by others or ratings of “leaderlike-ness”. Studies also vary in the extent of formality-informality of leadership roles.

Concerned with the context-free/blind nature of positivist psychological research, we wondered: What is the range of research interests on LE today? We were particularly interested in studies examining leadership emergence in real world social settings. We are thus delighted to curate nine papers for this Research Topic with a mix of conceptual and empirical papers (including quantitative, qualitative and mixed-methods studies). In this Research Topic, Popper provides evolutionary, psychodynamic and social psychological arguments for why leader emergence occurs naturally.

Taking a more agentic approach to LE (cf. Aycan and Shelia, 2019), Kennedy et al. show how motivations to lead mediate the relationship between the bright and dark personality traits and leadership intention. Auvinen et al. show the impact of leaders' motivation to lead on follower well being. Karakulak et al. explore gender differences in opt-out and push-out processes in LE from the lenses of emotions (i.e., worries about leading).

Focusing on processes underlying LE, Chang et al. show how values-based leadership is unlikely to emerge if left to natural processes or chance; they suggest mechanisms and boundary conditions for values-based leadership to emerge. Samdanis and Lee show how the processes of achievement and ascription can help us appreciate creative leader emergence in the social network context of “Art Worlds”. Bracht et al. focus on social learning processes involved in individual leader development, which is one context in which LE occurs. Qualitative research by Myeza and April explains how the motivation NOT to lead among Black professionals in post-Apartheid South Africa is shaped by specific historical, societal, generational, inter-racial group contexts. Finally, Ozcan reminds us that leadership is socially-constructed and is not only about the leader's traits and qualities; that cultural meanings/mental models of leadership shape the kind of leader-centered or collective leadership that emerges in social organizations.

Together, these papers echo many of the fundamental lessons learned from academic and practical interest in “why LE matters and what is wrong with it”. They remind us of the limitations of our conceptualizations, theoretical lenses, methodological approaches and de-contextualized treatment of issues in real world contexts. This Research Topic also inspires us to investigate the pathways to emergence of fine leaders and avoidance of flawed ones.

## Author Contributions

KC contributed to the writing of the editorial. ZA, MO, and KC contributed equally to the idea generation and conceptualization of the editorial. All authors contributed to the article and approved the submitted version.

## Conflict of Interest

The authors declare that the research was conducted in the absence of any commercial or financial relationships that could be construed as a potential conflict of interest.

## Publisher's Note

All claims expressed in this article are solely those of the authors and do not necessarily represent those of their affiliated organizations, or those of the publisher, the editors and the reviewers. Any product that may be evaluated in this article, or claim that may be made by its manufacturer, is not guaranteed or endorsed by the publisher.

## References

[B1] Acton B. P. Foti R. J. Lord R. G. Gladfelter J. A. (2019). Putting emergence back in leadership emergence: a dynamic, multilevel, process-oriented framework. Leadership Q. 30, 145–164. 10.1016/j.leaqua.2018.07.002

[B2] Aycan Z. Shelia S. (2019). “Leadership? no, thanks!” a new construct: worries about leadership. Eur. Manage. Rev. 16, 21–35. 10.1111/emre.12322

[B3] Hanna A. A. Smith T. A. Kirkman B. L. Griffin R. W. (2021). The emergence of emergent leadership: a comprehensive framework and directions for future research. J. Manage. 47, 76–104. 10.1177/0149206320965683

[B4] Harms P. D. Wood D. Landay K. Lester P. Vogelgesang-Lester G. (2018). Autocratic leaders and authoritarian followers revisited: a review and agenda for the future. Leadership Q. 29, 105–122. 10.1016/j.leaqua.2017.12.007

[B5] Hogan R. Curphy G. J. Hogan J. (1994). What we know about leadership: effectiveness and personality. Am. Psychol. 49, 493. 10.1037/0003-066X.49.6.493 8042818

[B6] Hollander E. P (1961). “Emergent leadership and social influence,” in Leadership and Interpersonal Behavior, eds L. Petrullo and B. M. Bass (New York: Holt, Rinehart & Winston), 30–47.

[B7] Kakkar H. Sivanathan N. (2017). When the appeal of a dominant leader is greater than a prestige leader. Proc. Natl. Acad. Sci. 114, 6734–6739. 10.1073/pnas.1617711114 28607061 PMC5495227

[B8] Lord R. G. De Vader C. L. Alliger G. M. (1986). A meta-analysis of the relation between personality traits and leadership perceptions: an application of validity generalization procedures. J. Appl. Psychol. 71, 402. 10.1037/0021-9010.71.3.402

[B9] Northway M (1946). Sociometry and some challenging problems of social relationships. Sociometry 9, 187–198. 10.2307/278500418476479

[B10] Samdanis M. Özbilgin M. (2020). The duality of an atypical leader in diversity management: the legitimization and delegitimization of diversity beliefs in organizations. Int. J. Manage. Rev. 22, 101–119. 10.1111/ijmr.12217

